# Analysis of the association of long working hours on anxiety and self-reported work-related musculoskeletal symptoms among female workers in electronics manufacturing enterprises in Guangdong Province

**DOI:** 10.3389/fpubh.2026.1892281

**Published:** 2026-07-31

**Authors:** Xiaoyi Li, Yushuo Liang, Huiqing Chen, Xiqing Liao, Xinyang Yu, Lin Xu, Junle Wu, Jiabin Chen, Min Yang

**Affiliations:** Guangdong Province Hospital for Occupational Disease Prevention and Treatment, Guangzhou, China

**Keywords:** anxiety, electronics manufacturing enterprise, female worker, long working hours, positive association, self-reported work-related musculoskeletal symptoms

## Abstract

**Background:**

Female workers were the main force in electronic manufacturing enterprises. This study aims to understand the current status of long working hours among female workers in the electronic manufacturing industry in Guangdong Province, and to explore the association of long working hours on anxiety and self-reported work-related musculoskeletal symptoms.

**Methods:**

We conducted a cross-sectional survey using convenience sampling method from July to September 2022, including a total of 193 electronic manufacturing enterprises in Guangdong Province. We distributed a total of 5,300 copies of the *Individual questionnaire of the National Key Population Occupational Health Literacy Monitoring Survey*, which included general demographic characteristics, and the detection rate of long working hours, anxiety and self-reported work-related musculoskeletal symptoms. A total of 4,976 female workers participated in the questionnaire survey after excluding invalid questionnaires, with the effective rate of 93.9%. A binary logistic regression analysis was used to evaluate the association of weekly working hours on anxiety and self-reported work-related musculoskeletal symptoms.

**Results:**

The detection rates of long working hours (working more than 40 h a week), anxiety and self-reported work-related musculoskeletal symptoms among female workers in electronics manufacturing enterprises in Guangdong Province were 67.8%, 23.0%, and 43.2%, respectively. The detection rate of self-reported work-related musculoskeletal symptoms in the anxiety group was higher than that in the non-anxiety group (65.2 *vs*. 36.7%, *P* < 0.001). The logistic regression model showed that long working hours were positively correlated with anxiety and self-reported work-related musculoskeletal symptoms after controlling for factors such as age, education level, marital status, length of service, enterprise scale, enterprise nature and night shift (both *P* < 0.01). Specifically, the longer weekly working hours exhibited a positive association with the development of anxiety and self-reported work-related musculoskeletal symptoms, with *OR* (95%*CI*) values of 1.067 (1.021–1.116) and 1.067 (1.028–1.108), respectively.

**Conclusions:**

The female workers employed in electronics manufacturing enterprises in Guangdong Province generally worked for long weekly hours, and a positive association was found between long working hours and the occurrence of anxiety and self-reported work-related musculoskeletal symptoms.

## Background

With the ongoing process of globalization and great changes in the working environment, the phenomenon of long working hours among working groups has become increasingly common, particularly in the manufacturing and internet industries (1, 2). Long working hours are defined as working beyond the standard limits — exceeding 8 h per day or 40 h per week (3). According to the International Labor Organization, nearly 500 million workers worldwide work more than 55 h per week (4). As the world's second-largest economy, China's rapid economic and social development had been accompanied by increased work demands across various industries, with the weekly working hours of employees approaching 48 h (5). Prolonged working hours not only reduce workers' personal time, but also lead to serious physical and mental health issues, making it one of the most critical occupational risk affecting workers' wellbeing (6, 7).

In recent years, self-reported work-related musculoskeletal symptoms have become one of the key occupational health issues in China (8, 9). It affects a wide range of industries and populations, and in addition to the physiological impact on workers, it would also reduce work efficiency and quality of life, and cause economic burdens to both society and individuals (10, 11). Consistent with existing epidemiological consensus, self-reported work-related musculoskeletal symptoms were recognized as multifactorial outcomes jointly shaped by occupational and non-occupational risk factors, whose interactions created confounding effects on physical discomfort severity and anxiety status (12–14). Previous studies showed that long working hours were associated with adverse health outcomes such as anxiety and self-reported work-related musculoskeletal symptoms (15, 16). Anxiety refers to the undesirable emotional state experienced by an individual in anticipation of difficulties or danger (17). It is a clinically common, chronic, and recurrent psychiatric disorder characterized by a general sense of nervousness, fear, restlessness, and distress (18).

Our country established *the Occupational Health Literacy Questionnaire of National Key Populations* in 2022, which was the first time to carry out a nationwide monitoring survey on occupational health literacy of key populations (19). One of the contents was to monitor and investigate the occupational health literacy of key populations in the country (20). In China, manufacturing accounts for the largest proportion of the workers, with the electronics manufacturing industry being a particularly prominent sector (21). As one of the leading industries in Guangdong Province, the electronics manufacturing industry has become an important strategic industry to promote the rapid economic development of Guangdong Province. Female workers are the main force in electronics manufacturing enterprises, and they not only take the family responsibility of giving birth to children, but also participate in production activities (22). Due to the characteristics of the industry, female workers in the electronics manufacturing industry require to work for long hours performing repetitive, monotonous, and fast-paced operations, which result in prolonged physical fatigue. This persistent fatigue may lead injuries in the waist, neck and shoulders, and other parts, subsequently causing absenteeism, production accidents, and reduce work ability. These outcomes may lead to indirect economic losses, which was not conducive to the sustainable development of enterprises and even the social economy (23, 24).

In accordance with the relevant requirements of the *Occupational Health Literacy Questionnaire of National Key Populations*, combined with the characteristics of the industrial structure of Guangdong Province, this study investigates the health association caused by long working hours of female workers in electronic manufacturing enterprises in Guangdong Province, aims to evaluate the association of long working hours on the anxiety and self-reported work-related musculoskeletal symptoms of female workers in electronics manufacturing enterprises in Guangdong Province, and proposes key strategies for the prevention and control of the health effects caused by long working hours, and provides new ideas for promoting the physical and mental health of this occupational population.

## Methods

### Subjects

This study adopted convenience sampling to recruit 193 electronics manufacturing enterprises across the Pearl River Delta, eastern, western and northern Guangdong, covering factories of various sizes and ownership types. Eligible enterprises had operated normally for over 12 months with repetitive assembly production lines and at least 30 frontline female workers. All qualified enterprises received research invitations without selective elimination, and all finished questionnaire collection. All female frontline workers satisfying individual inclusion criteria (aged ≥18 years, with over 6 consecutive months of service in the factory) were fully invited. Investigators introduced the survey in workshops, and all eligible workers could voluntarily scan QR codes to fill questionnaires, with the right to refuse or quit midway. A total of 5,300 questionnaires were distributed originally. After double-data entry and verification, questionnaires were defined as invalid and excluded if meeting any criteria: over 50% missing core items about weekly working hours, GAD-7 anxiety scale and 9-part musculoskeletal scale; logically contradictory answers on age, service length or working hours; identical scores filled for all scale items; completion time less than 90 s. Even fully completed questionnaires were excluded from the final sample of 4,976 participants if respondents met exclusion criteria: suffering severe negative emotional events in the latest 3 months, mental disorders or chronic organic diseases; previously diagnosed with osteoarthritis, bone tumors, joint tuberculosis, or congenital/traumatic musculoskeletal injuries interfering with self-reported work-related musculoskeletal symptoms evaluation. Researchers first screened risky subjects via questionnaires, and conducted brief on-site inquiries for ambiguous information. Questionnaires from disqualified respondents were discarded, with a valid response rate of 93.9%.

### Ethics approval and consent to participate

The present study was approved by the *Medical Ethics Committee of the Guangdong Province Hospital for Occupational Disease Prevention and Treatment* (Approval No. GDHOD MEC 2023075) and was strictly compliant with local law and the Declaration of Helsinki. All participants provided informed consent prior to administering the survey.

### Study design and population

The *Occupational Health Literacy Questionnaire of National Key Populations* was used to investigate the sociodemographic characteristics of the research subjects, such as age, gender, education level, marital status, personal monthly income, length of service, enterprise scale, enterprise nature, night shift, weekly working hours and other occupational characteristics (20). The classification of enterprise size was based on *the Measures for the Classification of Large, Medium, Small, and Micro Enterprises in Statistics* (*2017*), which categorizes enterprises as large- (≥1,000 employees), medium- (300– < 1,000 employees), and small- and micro-sized (< 300 employees) (25). Long working hours detection rate (%) = (number of participants working >40 h/week/total population) × 100%.

### Anxiety

The anxiety survey of the Occupational Health Literacy Questionnaire of National Key Populations used the generalized anxiety scale developed by Spitzer et al. and translated by He et al. (26, 27) among Chinese general hospital outpatients. The validation study confirmed good reliability and validity of the Chinese GAD-7, supporting its reliable application in domestic occupational population surveys. The scale consists of seven items, and the score was calculated based on the frequency of anxiety symptoms over the past two weeks: 0 points for “not at all”, 1 point for “several days”, 2 points for “more than a week”, and 3 points for “almost every day”. The total score was calculated as the sum of seven items, ranging from 0 to 21. A total score of 0–4 points indicated no anxiety, while 5–21 points indicated the presence of anxiety. A higher total score indicated a higher level of anxiety. Anxiety detection rate (%) = (number of participants with anxiety/total population) × 100%. The Cronbach's α coefficient for the scale in this study was 0.934, reflecting excellent internal consistency.

### Work-related musculoskeletal disorders

*The Musculoskeletal Disorders Survey Scale* of the *Occupational Health Literacy Questionnaire of National Key Populations* was developed by Yang et al., a domestic self-developed scale validated for Chinese manufacturing workers. Previous psychometric tests verified its acceptable reliability and discriminative validity for screening occupational musculoskeletal pain across nine body sites: neck, shoulder, back, elbow, waist, wrist, hip, hip, knee and ankle and foot (28). For the purpose of this study, an ache, pain or stiffness lasting for over 24 h within the past 12 months was defined as work-related if the symptom onset, aggravation or persistence was temporally linked to repetitive assembly line operations, prolonged static postures, frequent upper limb movements, long standing/sitting shifts, or heavy repetitive handling during daily production tasks. Any musculoskeletal discomfort triggered or worsened by off-job activities (housework, long hours of mobile phone use, recreational sports, chronic underlying orthopedic disease, age-related joint degeneration, sleep posture problems) was classified as non-work-related musculoskeletal symptoms. For each body part listed above, if work-related symptoms occurred within the past year and lasted for more than 24 h, a score of 1 was assigned; if no pain or discomfort was reported, a score of 0 was assigned. The total score was calculated as the sum of scores for nine body parts. A total score of 0 indicated no self-reported work-related musculoskeletal symptoms, while a score of 1–9 indicated the presence of self-reported work-related musculoskeletal symptoms. Higher total scores indicated a more severe self-reported work-related musculoskeletal symptoms. Self-reported work-related musculoskeletal symptoms detection rate (%) = (number of participants with self-reported work-related musculoskeletal symptoms at any site/total population) × 100%. The Cronbach's α coefficient for the scale in this study was 0.884, showing satisfactory internal consistency.

### Quality control

A full-process standardized quality control framework with quantitative quality parameters was adopted in this cross-sectional survey to reduce measurement bias and guarantee data reliability, covering investigator training, online questionnaire design, invalid sample screening, dual data entry and on-site rechecking.

First, unified standardized training was compulsory for all field investigators, with clear assessment parameters. Training contents included survey background, standardized respondent guidance, privacy protection protocols and unified invalid questionnaire judgment criteria. Investigators must pass a simulation assessment with a score ≥90 out of 100 before conducting field surveys to avoid inconsistent verbal explanations that may interfere with participants' answers.

Second, the QR-based online questionnaire system embedded rigid filling constraints as core quality control parameters. All demographic variables, weekly working hours, GAD-7 anxiety scale and nine-part musculoskeletal disorder scale items were set as mandatory non-skippable questions to avoid massive missing core data. A minimum completion time threshold of 90 s was preset; any questionnaire finished within less than 90 s was automatically marked as low-quality and eliminated to exclude random perfunctory filling.

Third, uniform quantitative criteria were formulated to screen invalid questionnaires after data collection. The key screening parameters included three dimensions: missing value threshold, logical contradiction and repetitive uniform responses. Questionnaires with over 50% missing items on core indicators (working hours, anxiety and musculoskeletal scale items) were excluded. Samples containing contradictory logic between age, length of service and weekly working hours were discarded. Records with identical scores across all scale items were also identified as invalid due to response bias.

Fourth, standardized database entry and auditing parameters were applied via EpiData 3.0. Value range limits were pre-set for all variables, such as restricting participants' age between 18 and 60 years old and limiting scale scores within official scoring ranges. Two independent researchers completed double entry of all questionnaires, with a 100% consistency requirement. Any inconsistent records between two datasets were cross-referenced with original electronic questionnaires and manually corrected to eliminate typing errors.

Finally, supplementary on-site verification was arranged for ambiguous records screened in preliminary data sorting, including extreme weekly working hour values and abnormally high anxiety scale scores to confirm response authenticity and correct misreported information.

In field implementation, trained investigators introduced survey purposes, filling requirements and confidentiality commitments to participants to ensure full comprehension of questionnaire rules. All respondents finished questionnaires by scanning exclusive QR codes and could not submit until all items were completed. The dual-entry verification mechanism further ensured the integrity and accuracy of the final analytical dataset.

### Statistical analysis

Data were analyzed using SPSS 21.0 software. Normally distributed measurement data were described using mean±standard deviation, while non-normally distributed data were described using median (*M*) and the 25th and 75th percentiles (*P*_25_, *P*_75_). The comparison of rates of count data was conducted using Pearson's Chi-square test or the trend Chi-square test. The association of weekly working hours on anxiety and self-reported work-related musculoskeletal symptoms was analyzed using binary logistic regression analysis. Weekly working hours were treated as an ordinal variable coded from 1 to 5 ( ≤ 40 = 1, 41–44 = 2, 45–48 = 3, 49–54 = 4, ≥55 = 5) in the primary model; an alternative categorical model with ≤ 40 h as the reference group was also tested for robustness. All variables with significant differences (*P* < 0.05) in univariate analysis were included in the adjusted model. Variable assignments were listed in Table 1. The Hosmer–Lemeshow test was applied to evaluate the goodness-of-fit of the regression model, and multicollinearity test was conducted to assess collinearity among independent variables. The significance level was set at α = 0.05.

**Table 1:** Variable assignment situation.

Variable	Assignment situation
Anxiety	No = 0, Yes = 1 (dependent variable)
Self-reported work-related musculoskeletal symptoms	No = 0, Yes = 1 (dependent variable)
Age	18–25 = 1, 26–35 = 2, 36–45 = 3, 46–60 = 4 (ordinal variable)
Education level	Junior high school and below = 1, High school/Technical secondary school = 2, college degree and above = 3 (ordinal variable)
Marital status	Married = 1, unmarried/divorced/widowed = 2 (categorical variable, reference group: married)
Personal monthly income	< ¥ 3,000 = 1, ¥ 3,000-¥ 4,999 = 2, ¥ 5,000-¥ 6,999 = 3, ≥¥ 7,000 = 4 (ordinal variable)
Length of service (years)	0.5– < 2.0 = 1, 2.0– < 5.0 = 2, 5.0– < 10.0 = 3, 10.0– < 20.0 = 4, 20.0–34.0 = 5 (ordinal variable)
Enterprise scale	Micro = 1, Medium = 2, Large = 3 (ordinal variable)
Enterprise nature	State-owned = 1, Private sector = 2, Foreign capital = 3 (categorical variable, reference group: state-owned)
Night shift	No = 0, Yes = 1 (categorical variable, reference group: No)
Weekly working hours	Ordinal variable coding: ≤ 40 = 1, 41–44 = 2, 45–48 = 3, 49–54 = 4, ≥55 = 5 (entered into the regression model as 1–5 ordinal codes) Categorical variable option: 5 categories with ≤ 40 h as the reference group

## Results

### Basic information

A total of 4,976 participants were included in the study, with a mean age of 36.4 ± 7.8 years. The median (*P*_25_, *P*_75_) of length of service was 3 (1, 8) years. Most participants were aged from 36.0 to < 46.0 years (39.7%), had a junior high school education or below (59.6%), and were married (83.6%) without night shifts (75.7%). The monthly average income was primarily between 3,000 and 4,999 yuan (55.0%), and the length of service was mostly 0.5– < 2 years (30.9%). Regarding enterprise size and characteristics, small- and micro-enterprises accounted for 44.5%, privately-owned enterprises accounted for 51.1%.

### Comparison of the detection rate of long working hours, anxiety, and self-reported work-related musculoskeletal symptoms among research subjects in different characteristic groups

A total of 3,375 (67.8%) study subjects had long working hours. A total of 1,146 study subjects were identified as anxiety, while 2,151 were screened as self-reported work-related musculoskeletal symptoms, with a detection rate of 23.0% (1,146/4,976) and 43.2% (2,151/4,976), respectively. The detection rate of long working hours among study subjects differed significantly by monthly income, enterprise size, and enterprise nature (all *P* < 0.01). The detection rate of anxiety among the study subjects with different ages, education level, marital status, length of service, enterprise size, night shift and weekly working hours showed significant difference (all *P* < 0.05). There were significant differences in the detection rate of self-reported work-related musculoskeletal symptoms among the study subjects with different length of service, enterprise size, enterprise nature, night shift and weekly working hours (all *P* < 0.05). See Table 2.

**Table 2:** Comparison of the detection rate of long working hours, anxiety and self-reported work-related musculoskeletal symptoms among study subjects in different groups.

Groups	Total, n (%)	Long working hours, *n* (%)	Anxiety, *n* (%)	Self-reported work-related musculoskeletal symptoms, *n* (%)
Age				
18–25	472 (9.5)	306 (64.8)	156 (33.1)	200 (42.4)
26–35	1,852 (37.2)	1,262 (68.1)	506 (27.3)	781 (42.2)
36–45	1,976 (39.7)	1,362 (68.9)	382 (19.3)	884 (44.7)
46–60	676 (13.6)	445 (65.8)	102 (15.1)	286 (42.3)
χ^2^-value	–	0.103	83.285	0.532
*p*-value	–	0.748	< 0.001	0.466
Education level				
Junior high school and below	2,965 (59.6)	2,002 (67.5)	614 (20.7)	1,263 (42.6)
High school/technical secondary school	1,201 (24.1)	842 (70.1)	306 (25.5)	521 (43.4)
College degree and above	810 (16.3)	531 (65.6)	226 (27.9)	367 (45.3)
χ^2^-value	–	0.141	23.283	1.812
*p*-value	–	0.707	< 0.001	0.178
Marital status				
Married	4,158 (83.6)	2,830 (68.1)	886 (21.3)	1,791 (43.1)
Unmarried/divorced/widowed	818 (16.4)	545 (66.6)	260 (31.8)	360 (44.0)
χ^2^-value	–	0.646	42.322	0.244
*p*-value	–	0.422	< 0.001	0.621
Personal monthly income				
< ¥ 3,000	776 (15.6)	420 (54.1)	159 (20.5)	330 (42.5)
¥ 3,000–¥ 4,999	2,737 (55.0)	1,882 (68.8)	628 (22.9)	1,214 (44.4)
¥ 5,000–¥ 6,999	1,197 (24.1)	883 (73.8)	296 (24.7)	490 (40.9)
≥¥ 7,000	266 (5.3)	190 (71.4)	63 (23.7)	117 (44.0)
χ^2^-value	–	62.051	3.742	0.460
*p*-value	–	< 0.001	0.053	0.498
Length of service (years)				
0.5– < 2.0	1,536 (30.9)	1,043 (67.9)	392 (25.5)	631 (41.1)
2.0– < 5.0	1,293 (26.0)	889 (68.8)	307 (23.7)	568 (43.9)
5.0– < 10.0	1,120 (22.5)	750 (67.0)	214 (19.1)	472 (42.1)
10.0– < 20.0	853 (17.1)	581 (68.1)	194 (22.7)	394 (46.2)
20.0–34.0	174 (3.5)	112 (64.4)	39 (22.4)	86 (49.4)
χ^2^-value	–	0.378	6.596	6.284
*p*-value	–	0.538	0.010	0.012
Enterprise scale				
Micro-	2,215 (44.5)	1,384 (62.5)	440 (19.9)	864 (39.0)
Medium-	1,345 (27.0)	977 (72.6)	300 (22.3)	574 (42.7)
Large-	1,416 (28.5)	1,014 (71.6)	406 (28.7)	713 (50.4)
χ^2^-value	–	52.547	38.350	45.541
*p*-value	–	< 0.001	< 0.001	< 0.001
Enterprise nature				
State-owned	66 (1.3)	42 (63.6)	20 (30.3)	51 (77.3)
Private sector	2,544 (51.1)	1,783 (70.1)	576 (22.6)	1,020 (40.1)
Foreign capital	2,366 (47.5)	1,550 (65.5)	550 (23.2)	1,080 (45.6)
χ^2^-value	–	12.296	2.248	46.990
*p*-value	–	0.002	0.325	< 0.001
Night shift				
No	3,767 (75.7)	2,554 (67.8)	816 (21.7)	1,535 (40.7)
Yes	1,209 (24.3)	821 (67.9)	330 (27.3)	616 (51.0)
χ^2^-value	–	0.005	16.386	38.821
*p*-value	–	0.944	< 0.001	< 0.001
Weekly working hours				
≤ 40	1,601 (32.2)	–	354 (22.1)	647 (40.4)
41–44	857 (17.2)	–	179 (20.9)	369 (43.1)
45–48	874 (17.6)	–	176 (20.1)	337 (38.6)
49–54	619 (12.4)	–	155 (25.0)	291 (47.0)
≥55	1,025 (20.6)	–	282 (27.5)	507 (49.5)
χ^2^-value	–	–	11.260	20.729
*p-*value	–	–	0.001	< 0.001

### Detection rate of self-reported work-related musculoskeletal symptoms among study subjects in different anxiety groups

The detection rates of self-reported work-related musculoskeletal symptoms among study subjects had significantly difference (χ^2^ = 292.46, *P* < 0.001) between the non-anxiety group (36.7%; 1,404/3,830) and the anxiety group (65.2%; 747/1,146). Among them, the detection rate of self-reported work-related musculoskeletal symptoms for 1–2 sites in the non-anxiety group was higher than that in the anxiety group, while the detection rates of self-reported work-related musculoskeletal symptoms for 3–4 sites and more than 5 sites in the anxiety group were both higher than those in the non-anxiety group. See Table 3.

**Table 3:** Prevalence of self-reported work-related musculoskeletal symptoms stratified by anxiety status and number of affected body sites.

Groups	Total participants (*n*)	Any part, *n* (%)	1–2 sites, *n* (%)	3–4 sites, *n* (%)	More than 5 sites, *n* (%)
Non-anxiety	3,830	1,404 (36.7)	695 (49.5)	398 (28.3)	311 (22.2)
Anxiety	1,146	747 (65.2)	223 (29.9)	223 (29.9)	301 (40.3)
Total	4,976	2,151 (43.2)	918 (42.7)	621 (28.9)	612 (28.5)

### Analysis of the association of weekly working hours on both anxiety and self-reported work-related musculoskeletal symptoms

Binary logistic regression analyses were conducted with anxiety and self-reported work-related musculoskeletal symptoms as dependent variables. Weekly working hours were the primary predictor, along with other variables that showed significant difference (*P* < 0.05) in univariate analysis (Table 2). The results showed that after controlling for age, education level, marital status, length of service, enterprise size, enterprise nature, night shift and other factors, the weekly working hours were positively correlated with both anxiety and self-reported work-related musculoskeletal symptoms (all *P* < 0.01). Specifically, for each one higher category of weekly working hours (coded 1–5), the odds of developing anxiety increased by 6.7% (*OR* = 1.067, 95%*CI* 1.021–1.116, *P* = 0.004), and the odds of developing self-reported work-related musculoskeletal symptoms increased by 6.7% (*OR* = 1.067, 95%*CI* 1.028–1.108, *P* = 0.001). This *OR* value reflects the change per category increase, not per additional hour of work, as the working-hour categories are not equal intervals. The full results of the adjusted model are shown in Table 4.

**Table 4:** Binary logistic regression analysis of the association of weekly working hours on anxiety and self-reported work-related musculoskeletal symptoms.

Variable	β value	*SE* value	Wald χ^2^ value	*P* value	*OR (95%CI*) value
Anxiety model					
Weekly working hours (ordinal, 1–5)	0.065	0.023	8.270	0.004	1.067 (1.021–1.116)
Age	0.021	0.011	3.545	0.060	1.021 (0.999–1.043)
Education level	0.087	0.032	7.391	0.007	1.091 (1.024–1.162)
Marital status (vs. married)	−0.125	0.048	6.781	0.009	0.883 (0.804–0.970)
Length of service (years)	0.032	0.015	4.551	0.033	1.033 (1.003–1.064)
Enterprise scale	0.112	0.028	16.000	< 0.001	1.119 (1.059–1.182)
Enterprise nature (vs. state-owned)					
Private sector	0.058	0.062	0.871	0.351	1.060 (0.939–1.196)
Foreign capital	0.076	0.063	1.455	0.228	1.079 (0.954–1.221)
Night shift (vs. No)	0.189	0.042	20.250	< 0.001	1.208 (1.113–1.311)
Self-reported work-related musculoskeletal symptoms model					
Weekly working hours (ordinal, 1–5)	0.065	0.019	11.544	0.001	1.067 (1.028–1.108)
Age	0.018	0.009	4.000	0.045	1.018 (1.000–1.036)
Education level	0.056	0.026	4.629	0.031	1.058 (1.005–1.114)
Marital status (vs. married)	−0.089	0.039	5.204	0.023	0.915 (0.848–0.987)
Length of service (years)	0.041	0.012	11.674	0.001	1.042 (1.018–1.067)
Enterprise scale	0.156	0.023	46.099	< 0.001	1.169 (1.118–1.223)
Enterprise nature (vs. state-owned)					
Private sector	−0.123	0.050	6.052	0.014	0.884 (0.801–0.976)
Foreign capital	−0.098	0.051	3.699	0.054	0.907 (0.820–1.003)
Night shift (vs. No)	0.215	0.034	40.062	< 0.001	1.240 (1.160–1.325)

## Discussion

In recent years, the “996” (9 a.m.−9 p.m., 6 days a week) and even “007” (without work shifts) working models have led to physical exhaustion and increasing psychological problems among workers, sparking widespread public concern about long working hours (6, 29). In this study, the detection rate of long working hours of female workers in electronics manufacturing enterprises in Guangdong Province reached 67.8%, which is comparable to the 61.1% reported by Zhu et al. (30) in power supply enterprises and much higher than the 42.7% reported by Li (31) in the petrochemical industry. Although longer working hours may appear to improve productivity, it can cause the long-term negative impacts, including the loss of personal time, job burnout, depressive symptoms, self-reported work-related musculoskeletal symptoms, and sleep disorders, all of which ultimately reduce work efficiency and quality (15, 32, 33). Weekly working hours showed a positive graded correlation with both anxiety and musculoskeletal symptomatic complaints. With the extension of weekly working hours from ≤ 40 h to ≥55 h, anxiety detection rate rose gradually from 22.1 to 27.5%, and symptomatic complaint rate climbed from 40.4 to 49.5%, presenting an obvious upward trend (both *P* < 0.01). This descriptive gradient directly reflects that longer working hours correspond to higher risks of physical discomfort and negative psychological emotions. Therefore, a series of physical and mental health risks caused by long working hours to female workers in the electronics manufacturing enterprises warrant serious attention.

This study showed that night shift work had a synergistic correlation with adverse physical and mental outcomes. Workers with night shifts owned significantly higher anxiety (27.3 *vs*. 21.7%) and multi-site musculoskeletal symptom rates (51.0 *vs*. 40.7%) than day-shift employees, indicating night work could amplify the adverse health effects of long working hours. Enterprise scale was positively correlated with the two outcome indicators. The detection rates of anxiety and musculoskeletal symptoms increased sequentially from micro-, medium- to large-sized enterprises; large factories faced higher production output pressure, faster assembly line rhythm, and less flexible rest schedules, which jointly elevated physical and mental health risks. Length of service was correlated with the severity of musculoskeletal symptoms. Workers with longer service time (10–34 years) exhibited the highest proportion of multi-site symptomatic complaints, while short-service workers (0.5–2 years) had relatively mild symptomatic involvement; however, anxiety did not show a simple linear correlation with working years, suggesting that physical cumulative damage grows with service length, yet psychological anxiety is affected by more complex combined factors. Income level was closely correlated with long working hours. Employees with medium and high monthly income (¥ 3,000–¥ 7,000) had far higher overtime exposure rates than low-income groups (< ¥ 3,000), revealing that extra income was mostly obtained through extended working time, which further mediated the correlation between income and physical and mental discomfort.

WMSDs are prevalent across various industries and populations with a high prevalence, representing a major occupational health challenge in China and a global concern in occupational health (34, 35). In Southeast Asia, the prevalence of WMSDs has been reported as 78.3% in Thailand, 81.3% in Indonesia, and 88.4% in Malaysia (36). A cross-sectional study in India has indicated that the 12-month prevalence of WMSDs was 75.8% and the point prevalence was 23.3% among dentists (37). In eastern province of Saudi Arabia, the WMSDs prevalence among teachers was 41.1% (38), and in South Korea, WMSDs accounted for 9.5%−71.5% of the total number of occupational diseases annually, most commonly in the manufacturing industry, followed by the construction, transportation/warehousing, communication, and mining (11). In China, nearly all occupational groups are affected by WMSDs. Studies have shown that the overall WMSDs prevalence among manufacturing workers has reached 79.7%, often involving multiple body regions instead of just a single body part (39). Among local coal miners, the highest WMSDs prevalence among different body parts were the lower back (50.7%), followed by the neck, shoulders, knees, and elbows (40, 41). The prevalence among shipyard workers was 59.3% (42), while among ultrasound physicians, it reached 94.3%, with the right shoulder and neck being the most affected body parts (43). The prevalence of WMSDs among employees of Internet companies was as high as 80.4% (44). In the electronics manufacturing industry, workers are at increased risk of muscle and nerve injuries due to the nature of the work—prolonged sitting or standing, repetitive tasks, and forced postures, leading to fatigue and musculoskeletal pain (9, 45). Standard clinical WMSDs require standardized physical examination, imaging evidence and professional physician diagnosis to confirm pathological soft tissue or joint lesions. By contrast, this study only collected subjective pain, soreness and stiffness perceived by participants through self-report questionnaires, without clinical physical assessment or auxiliary examination. Therefore, the outcome indicator measured herein is self-reported work-related musculoskeletal symptoms, rather than clinically confirmed WMSDs. Most previous epidemiological studies in manufacturing industries adopted self-reported symptom scales as rapid screening tools, yet there exists an essential boundary between subjective symptomatic complaints and clinical WMSDs confirmed by medical examination. Clinically defined WMSDs represent organic lesions including tenosynovitis, cervical spondylosis, lumbar intervertebral disc strain and shoulder soft tissue injury, which require objective clinical evidence for diagnosis. Self-reported symptoms only reflect workers' subjective sensory discomfort, which may be affected by pain tolerance, psychological sensitivity and recall bias, and cannot be equated to confirmed occupational diseases. In this study, the detection rate of self-reported work-related musculoskeletal symptoms among 4,976 female workers in 193 electronics manufacturing enterprises was 43.2%, which was slightly higher than the 39.5% found in previous study in two electronics manufacturing enterprises (46), but lower than the 58.0% in the electronics manufacturing industry in a city in the Pearl River Delta reported by Feng et al. (47). The high symptomatic detection rate in this sample reflects widespread physical discomfort complaints among female electronic workers, but does not represent the prevalence of clinically diagnosed WMSDs.

In addition to causing physiological risks, self-reported work-related musculoskeletal symptoms could also lead to a range of negative outcomes, such as loss of work ability, reduced work efficiency, lower income level, and lower quality of life, and increase the medical burden of society, causing huge economic burdens to both society and individuals (10, 11). A large number of epidemiological investigations had shown that the development of self-reported work-related musculoskeletal symptoms is related to multiple factors, including occupational factors, individual factors, and psychosocial factors (48, 49). According to a survey among workers in the automobile manufacturing industry, long working hours was a risk factor for self-reported work-related musculoskeletal symptoms (50), and similar findings were reported in studies of transportation workers (51). In this study, long working hours showed a positive association with self-reported work-related musculoskeletal symptoms, and each one higher category of weekly working hours was associated with a 6.7% increase in the odds of self-reported work-related musculoskeletal symptoms, which was consistent with previous study by our lab and by Wu et al. in employees from internet companies (52, 53). These findings suggested that rational work scheduling and limiting working hours may serve as effective strategies to prevent and intervene self-reported work-related musculoskeletal symptoms.

Both the Chinese GAD-7 anxiety scale and domestic WMSDs screening scale used in this research had undergone formal psychometric validation in prior Chinese population studies, and their high Cronbach's α coefficients in the current sample further confirmed stable reliability when applied to female frontline electronics manufacturing workers. In this study, the detection rate of anxiety among female workers in electronics manufacturing enterprises in Guangdong Province was 23.0%, which is slightly lower than the 26.8% reported by previous investigation in the Pearl River Delta region (54). Several studies had shown that long working hours is a risk factor for anxiety (16, 55). Anxiety is an undesirable emotional experience and an unhealthy psychological state, which not only negatively impacts workers' health, but also seriously reduces work efficiency, and imposes adverse effects on both individual wellbeing and society productivity (56). The electronics manufacturing industry is a leading industry in the economic development of Guangdong Province, facing an increasing competitive pressure nowadays. Female workers in this industry were more likely to suffer from psychological disorders such as anxiety and depression, since most of them also took family responsibilities such as childbirth and parenting (57). Demographic factors such as age, gender, body mass index, smoking, and physical exercise, as well as psychosocial factors including high mental stress, low social support, and high job satisfaction and job monotony were all played an important role in the development of self-reported work-related musculoskeletal symptoms (58). Stratified analysis by the number of affected body sites further revealed a clear gradient association between multi-site musculoskeletal lesions and anxiety status. For participants with mild self-reported work-related musculoskeletal symptoms involving only 1–2 body sites, the proportional distribution was higher among non-anxious workers (49.5 *vs*. 29.9% of all WMSD cases in each group). In contrast, respondents suffering from moderate (3–4 sites) and severe multi-site self-reported work-related musculoskeletal symptoms (≥5 sites) accounted for a larger share within the anxiety group (29.9 *vs*. 28.3% for 3–4 sites, 40.3 *vs*. 22.2% for ≥5 sites). This graded distribution indicates that mild single or dual-site musculoskeletal discomfort generates transient fatigue that workers can self-regulate through short rest breaks, resulting in minimal psychological interference. However, cumulative persistent discomfort across three or more body parts creates sustained physical stress, chronic sleep disturbance, impaired daily work performance, and work-family conflict. The superimposed physical discomfort and functional restriction jointly exacerbate psychological distress, raising the risk of clinically significant anxiety symptoms. It was suggested that enterprises should pay attention to the problem of female workers' long working hours, and adopted early intervention to improve work efficiency, reduce the incidence of anxiety, and reduced the association of self-reported work-related musculoskeletal symptoms, so as to promote the sustainable healthy development of enterprises and society. Given the cross-sectional descriptive design of this study, we could only observe correlated distribution trends rather than causal direction, yet the graded multi-site lesion pattern still provides meaningful descriptive evidence linking the severity of self-reported work-related musculoskeletal symptoms to elevated anxiety prevalence.

## Conclusion

In summary, female workers in electronics manufacturing enterprises in Guangdong Province generally worked for long working hours, which would increase the positive association of anxiety and self-reported work-related musculoskeletal symptoms. According to the relevant provisions of the *Labor Law of the People's Republic of China*, employers are required to ensure employees to have at least 1 day off per week, and that overtime hours should not exceed 1 h per day, or 3 h under special circumstances, with a monthly maximum of 36 h. Based on the results of this study, following recommendations are proposed for enterprises. (i) Utilizing technological support. Implementing intelligent equipment and software tools in automate repetitive and labor-intensive processes, thereby reduce the working hours for female employees (59). (ii) Optimizing personnel allocation. Encourage rotational leave during off-peak seasons; hiring temporary workers or reallocating internal workers during peak production periods, to reduce the frequency of prolonged work hours and protect workers' mental health (32). (iii) Adjusting compensation structures. Establish a flexible compensation system based on workload and seasonal variations. Distinguish between basic salaries and overtime pay, and provide performance-based incentives for workers engaged in prolonged hours. (iv) Enhancing training and communication. During off-peak seasons offer skill training to improve workers' professional competence and adaptability (60). Also, employers should be aware of workers' work status and emotional wellbeing, and respond to issues in time. As for female workers, the following suggestions are provided. (i) Developing reasonable work plans. Organize work schedules according to personal capacity to reduce unnecessary overtime (61). (ii) Strengthening professional skills. Improve personal technical skills to meet the demands of evolving industrial processes and enhance work efficiency (62). (iii) Improving health awareness. Adopt healthy work and lifestyle to prevent anxiety and self-reported work-related musculoskeletal symptoms associated with prolonged working hours (63).

This study focused on the association of long working hours on the anxiety and self-reported work-related musculoskeletal symptoms among female workers in electronic manufacturing enterprises. However, ergonomic and psychosocial factors were not included in the current analysis. In addition, as a cross-sectional study, it cannot infer causal relationships between long working hours and anxiety or self-reported work-related musculoskeletal symptoms. Future research would use a prospective cohort design to further explore the effects of adverse ergonomic factors, and provide more robust evidence for the prevention and treatment of anxiety and self-reported work-related musculoskeletal symptoms.

## Data Availability

The original contributions presented in the study are included in the article/supplementary material, further inquiries can be directed to the corresponding author.
